# IQ and Blood Lead from 2 to 7 Years of Age: Are the Effects in Older Children the Residual of High Blood Lead Concentrations in 2-Year-Olds?

**DOI:** 10.1289/ehp.7625

**Published:** 2005-02-02

**Authors:** Aimin Chen, Kim N. Dietrich, James H. Ware, Jerilynn Radcliffe, Walter J. Rogan

**Affiliations:** ^1^Epidemiology Branch, National Institute of Environmental Health Sciences, National Institutes of Health, Department of Health and Human Services, Research Triangle Park, North Carolina, USA;; ^2^Department of Environmental Health, University of Cincinnati, Cincinnati, Ohio, USA;; ^3^Harvard School of Public Health, Harvard University, Boston, Massachusetts, USA;; ^4^Department of Psychology, Children’s Hospital of Philadelphia, and University of Pennsylvania School of Medicine, Philadelphia, Pennsylvania, USA

**Keywords:** chelation, developmental testing, IQ, lead poisoning, longitudinal studies

## Abstract

Increases in peak blood lead concentrations, which occur at 18–30 months of age in the United States, are thought to result in lower IQ scores at 4–6 years of age, when IQ becomes stable and measurable. Data from a prospective study conducted in Boston suggested that blood lead concentrations at 2 years of age were more predictive of cognitive deficits in older children than were later blood lead concentrations or blood lead concentrations measured concurrently with IQ. Therefore, cross-sectional associations between blood lead and IQ in school-age children have been widely interpreted as the residual effects of higher blood lead concentrations at an earlier age or the tendency of less intelligent children to ingest more leaded dust or paint chips, rather than as a causal relationship in older children. Here we analyze data from a clinical trial in which children were treated for elevated blood lead concentrations (20–44 μg/dL) at about 2 years of age and followed until 7 years of age with serial IQ tests and measurements of blood lead. We found that cross-sectional associations increased in strength as the children became older, whereas the relation between baseline blood lead and IQ attenuated. Peak blood lead level thus does not fully account for the observed association in older children between their lower blood lead concentrations and IQ. The effect of concurrent blood level on IQ may therefore be greater than currently believed.

In 1994, two meta-analyses of the relationships between childhood lead exposure and IQ appeared (Pocock et al. 1994; Schwartz 1994). Both dealt with a problem that arises in the interpretation of prospective studies in which the child’s blood lead concentration peaks at about 2 years of age, but IQ can only be measured stably (with higher test–retest correlation) in children ≥4 years of age (Sternberg et al. 2001). Both meta-analyses focused on the strength of association between IQ at school age and either blood lead concentration measured at 2 years of age or derived measures of lead exposure, such as average blood lead up to 3 years of age, which was found to be correlated strongly with 2-year blood lead concentration.

One meta-analysis (Pocock et al. 1994) included cross-sectional studies in a separate analysis; those studies also showed an inverse association of blood lead concentration with IQ at ≥6 years of age. The other meta-analysis (Schwartz 1994) included cross-sectional studies in the overall model, but additional analysis of cross-sectional studies showed findings similar to those in the prospective studies. Both meta-analyses concluded that the prospective design was more reliable in studying blood lead–IQ associations, because reverse causality, in which impaired children ingest more leaded paint or dust, was less likely to produce an association. Neither meta-analysis emphasized the importance of cross-sectional associations in older children, and both implied that the interpretation most consistent with the overall results was that peak blood lead concentration, achieved at about 2 years of age, was most likely responsible for the cognitive effects, even though these effects were not reliably detectable until ≥ 4 years of age.

These meta-analyses as well as the findings from the individual prospective studies, especially Boston (Bellinger et al. 1992), have had a strong effect on the interpretation of both longitudinal and cross-sectional studies. One consequence is that if we wish to know the minimum concentration in blood at which lead affects cognition, then we are obliged to follow children starting at 2 years of age. School-age children, in whom IQ is relatively stable and testable, have lower blood lead concentrations. A cross-sectional study among school-age children might reflect latent damage done by higher blood lead concentrations at 2 years of age but detectable only at school age because IQ can be more reliably tested with greater precision at that age. This interpretation has guided screening programs, which focus on 1- and 2-year-olds; clinical trials, which have treated 2-year-olds; and the interpretation of the cross-sectional literature. Thus, the report in 2000 (Lanphear et al. 2000) that school-age children studied in the third National Health and Nutrition Examination Survey had an inverse relationship between their concurrent blood lead concentrations and IQ even at blood lead concentrations < 10 μg/dL, the current Centers for Disease Control and Prevention (CDC) level of concern, was interpreted by some to reflect the effects of the children’s higher blood lead concentrations when they were 2 years old. However, after publication of prospective data showing that even peak blood lead concentrations < 10 μg/dL in toddlers were associated with decrements in IQ (Canfield et al. 2003), changes in policy were quickly considered.

It is not clear that only the peak blood lead concentration matters. In the prospective Boston study that examined children with very low lead exposure, blood lead concentration at 2 years of age, but not at 57 months or 10 years of age, was significantly associated with IQ at 10 years of age (Bellinger et al. 1992). Other prospective studies of children with relatively high lead exposure, however, found concurrent (Dietrich 1995; Dietrich et al. 1993) or lifetime average blood lead concentration (Tong et al. 1996) to be more strongly associated with IQ measurement at school age than were peak levels. Another study modeling relative contribution of pre-natal and postnatal (0–2 years, 2 to 3–7 years) blood lead also found increases in blood lead after 2 years of age associated with IQ deficits after 2 years of age (Wasserman et al. 2000).

Other analyses have asked whether greater declines in blood lead concentration from the 2-year peak are associated with higher IQ later. Two studies with analyses that adjusted for 2-year cognitive test score found that children whose blood lead concentrations fell more after 2 years of age had increased IQ (Liu et al. 2002; Ruff et al. 1993). However, another study modeling change in IQ found that decline in blood lead concentration from 2 or 4 years of age to 11–13 years of age was not associated with IQ improvement, and that declines in blood lead concentration from 7 to 11–13 years of age were associated with only modestly better IQ scores (Tong et al. 1998). Overall, the findings suggest that the longer the blood lead is at high levels, the greater the effect on IQ, and thus they imply that blood lead concentration in school age may matter because higher concentrations usually reflect longer exposures or higher current body burdens as a result of heavy exposure in earlier years. It also raises the level of concern for children in areas where leaded gasoline and industrial sources are still a source of exposure. In general, outdoor exposures such as these produce peak blood lead concentrations at later ages among children (Wasserman et al. 1997). Although such exposure may be experienced by older and perhaps less susceptible children, this exposure might also be more prolonged and consequently produce more damage.

Here we examine a large prospective data set from a clinical trial of lead poisoning treatment. This data set includes blood lead concentrations measured frequently between entry at about 24 months of age and the last follow-up at 90 months, as well as the results of IQ testing at multiple times. We sought to clarify the strength of the association between IQ and blood lead at the various time points, to examine whether the cross-sectional associations seen in the 84- to 90-month-old children represent residual effects from 2 years of age or new effects emerging among these school-age children, and how the change in blood lead over time is related to IQ at follow-up.

## Materials and Methods

The Treatment of Lead-exposed Children (TLC) study was a multicenter, randomized, placebo-controlled clinical trial of 780 children 12–33 months of age with blood lead concentrations of 20–44 μg/dL. The study tested whether succimer treatment of toddlers resulted in better scores on IQ tests and other measures of behavioral and psychological function (TLC 1998). Although succimer lowered blood lead concentrations for about 10 months, it did not improve test scores at 36-month (Rogan et al. 2001) or 60-month follow-up (Dietrich et al. 2004). Thus, the succimer and placebo groups can be analyzed separately or combined to study the effect of blood lead concentration on cognitive test scores.

### Blood lead concentration.

Venous blood was collected with lead-free containers twice before randomization and on days 7, 28, and 42 after the beginning of each course of treatment. After the termination of treatment, blood lead concentrations were measured every 3–4 months. From up to 24 measurements of blood lead concentrations, we used the second blood sample before randomization at baseline (about 2 years of age), the blood sample at 36-month follow-up (about 5 years of age), and the last blood sample at 60-month follow-up (about 7 years of age) (TLC 2000). The peak blood lead concentration from baseline to 7 years of age was the maximum blood lead concentration of any of up to 24 measurements. For those having their blood lead concentration measured at 5 or 7 years of age, average blood lead concentration from baseline to 5 or 7 years of age was calculated by dividing the area under the blood lead concentration–age curve (inflecting a connecting line at each blood lead measurement, and then summing the areas of the trapezoids) by age interval. The blood lead concentrations were measured at the Nutritional Biochemistry Branch of the CDC by atomic absorption spectrometry based on the methods described by Miller et al. (1987).

### Cognitive test scores.

At baseline, children were given the Bayley Scales of Infant Development–II (BSID-II) (Bayley 1993), the most widely used scales of infant intelligence tests. The Mental Development Index (MDI) of BSID-II is statistically analogous to an IQ score. Like those from the Wechsler scales, standard scores from the BSID-II have a mean of 100 and standard deviation of 15. For all these measures, raw scores are transformed into these standard scores. The children’s full-scale IQ at 36-month follow-up was measured with the Wechsler Preschool and Primary Scales of Intelligence–Revised (Wechsler 1989), and at 60-month follow-up IQ was measured with the Wechsler Intelligence Scale for Children–III (Wechsler 1991). At one of the visits between enrollment and 36-month follow-up, the caregiver’s IQ (the mother for 88% of children, the father for 4%, and another caregiver for 8%) was measured with the two-subtest version of the Wechsler Adult Intelligence Scale–Revised (Silverstein 1985; Wechsler 1981).

### Statistical analysis.

We used multiple linear regression models to analyze the association of blood lead concentration and cognitive scores at various ages. Both log-transformed and untransformed blood lead concentrations were tested in the statistical models, and the findings were similar. In this article, only results of untransformed blood lead concentrations were used. Covariates include clinical center (Baltimore, Maryland; Cincinnati, Ohio; Newark, New Jersey; and Philadelphia, Pennsylvania), race (black or white/others), sex (male or female), language (English or Spanish), parent’s education (< 12 years, 12 years, > 12 years of education), parent’s employment (neither working, either working), single parent (yes or no), age at blood lead concentration test, and caregiver’s IQ.

First, one blood lead concentration (at 2, 5, and 7 years of age, peak or average) was included as an independent variable to model IQ score (MDI at 2 years, IQ at 5 or 7 years of age), with adjustment for covariates.

Then, to evaluate whether the cross-sectional association was a residue of the effect from an earlier blood lead concentration, both prior and concurrent blood lead concentration were included in the models (blood lead concentration at 2 and 5 years on IQ at 5 years of age, blood lead concentration at 2 and 7 years on IQ at 7 years of age, blood lead concentration at 5 and 7 years on IQ at 7 years of age). The model thus includes later IQ as the dependent variable, and earlier blood lead concentration, later blood lead concentration, and covariates as the independent variables. We also tried additional adjustment for earlier IQ, which theoretically tests for change in IQ by change in blood lead concentration. This allows us to compare results with previous studies using a similar approach (Liu et al. 2002; Ruff et al. 1993).

Quantitative analysis of both prior and concurrent blood lead concentration in the same model, even without adjustment for prior IQ, still faces the problem of collinearity between the two blood lead measurements. A qualitative alternative may reduce the precision in estimation but should show the effect on school-age IQ by blood lead concentration with less distortion. Therefore, we used the median of earlier blood lead concentration and the median of later blood lead concentration as cutoff points to reduce earlier and later blood lead concentrations to binary variables. This does not eliminate the inherent correlation between earlier and later blood lead concentrations (children with lower earlier blood lead concentrations tend to have lower later blood lead concentrations), but it reduces the impact of collinearity on the stability of the estimated regression coefficients and produces a model that is easy to interpret.

To see whether the association between blood lead concentration and IQ score varied by treatment group (succimer or placebo), we repeated these regression models by treatment group.

We used SAS (version 9.0; SAS Institute Inc., Cary, NC) for statistical analysis. All tests are two sided. Because of the difference in the number of children tested for each follow-up measurement, the sample sizes of the different regression models vary slightly.

## Results

### Study subjects.

Four centers were involved in the recruitment, treatment, and follow-up of a total of 780 children in the TLC study: Baltimore had 213, Cincinnati had 194, Newark had 208, and Philadelphia had 165. Three hundred ninety-six children were randomly assigned to the succimer group, and 384 to the placebo group. There were no differences between groups in age, sex, race, socioeconomic status, and blood lead concentration of children at recruitment. The children were mainly African American (77%), from households speaking English (95%), with a single parent (72%), and receiving public assistance (97%).

### Blood lead concentration and IQ scores.

The blood lead concentrations and IQ scores of TLC children at baseline and at 5 and 7 years of age are shown in Table 1. The correlation coefficients of blood lead concentrations at baseline and 5 or 7 years were 0.40 and 0.27, respectively; that between measurements at 5 and 7 years was 0.78. The correlation coefficients of cognitive scores at baseline and 5 or 7 years were 0.54 and 0.41, respectively; that between 5 and 7 years was 0.78. All of these correlation coefficients were statistically significant (all *p*-values < 0.001). Although mean blood lead concentration declined over time, the standard deviations remained similar. As shown in Table 1, the ages at IQ tests and blood lead concentration measurements were very close. For example, at about 7 years of age, 94% of children had IQ and blood lead tests on the same day.

### Heterogeneity between succimer and placebo groups.

There were no statistical differences between succimer and placebo groups in either blood lead concentrations or cognitive scores at the time points under consideration (all *p*-values > 0.10). The association between blood lead concentration and IQ score was homogeneous between treatment groups except for baseline blood lead concentration and baseline MDI: 10-μg/dL increment at baseline had a coefficient of –1.6 (SE, 1.3) MDI points in the succimer group and –4.6 (SE, 1.4) MDI points in the placebo group. In the regression models including both prior and concurrent blood lead concentrations without additional adjustment for prior IQ, the results were similar in the succimer and placebo groups.

### Associations of blood lead concentration and IQ.

**Figure 1:**
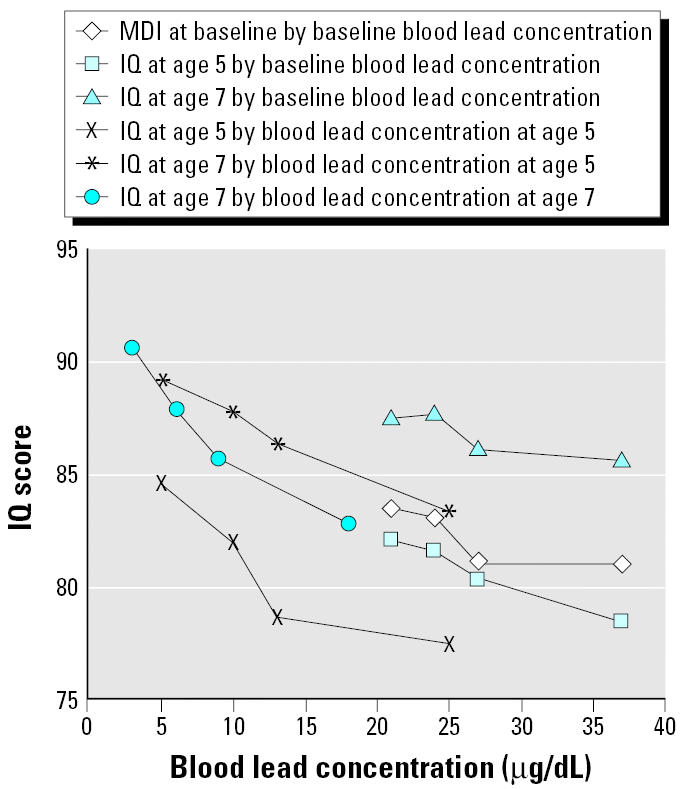
IQ test scores by prior or concurrent blood lead concentration. Each data point shows the mean IQ test scores of children measured at baseline or at two follow-ups, grouped by quartiles of blood lead concentration. The abscissa of each point is the middle value of each blood lead concentration category.

Figure 1 displays the mean IQ at current and subsequent ages by quartiles of blood lead measured at 2, 5, and 7 years of age. Although blood lead concentrations decreased over time and IQ test scores first declined and then increased, the inverse association between concurrent blood lead concentration and IQ test scores remained constant.

Table 2 shows the estimated difference in cognitive test scores for each 10-μg/dL change in prior, concurrent, peak, or average blood lead concentration. Overall, the concurrent blood lead concentration always had the strongest association with IQ, and as the children aged, the relationship grew stronger. The peak blood lead concentration from baseline to 7 years of age was not associated with IQ at 7 years of age. The average blood lead concentration from baseline to 5 or 7 years of age was inversely associated with IQ at 5 or 7 years of age.

### Is the cross-sectional association at a later age a residue of effects attributable to earlier blood lead concentrations?

In the model including both prior and concurrent blood lead concentrations, concurrent blood lead was always more predictive of later IQ scores (Table 3). Adjustment for prior IQ did not significantly change the strength of association with concurrent blood lead concentration. Alternatively, categorizing prior and concurrent blood lead concentrations by the corresponding medians in the regression model had similar results (Table 4). Among children with both prior and concurrent blood lead concentrations below the corresponding medians as the reference group, those with prior blood lead concentrations at or above median but concurrent blood lead concentrations below the median did not have a decrease in school-age IQ score. In contrast, children with concurrent blood lead concentrations at or above the median had roughly similar IQ decreases, irrespective of their prior blood lead concentration.

## Discussion

In these data we found a stronger relationship between blood lead concentration at 7 years and IQ at 7 years than between IQ at 7 years and the higher 2-year-old blood lead concentration; the association of blood lead concentration at 5 years and IQ at 5 years is also stronger than that between IQ at 5 years and blood lead at 2 years. The strength of the cross-sectional association increases over time (from 2 years of age to 5–7 years of age), despite lower blood lead concentrations in older children. This analysis supports the idea that lead exposure continues to be toxic to children as they reach school age, and does not support the interpretation that all the damage is done by the time the child is 2 or 3 years of age.

We found previously that children with relatively greater declines in blood lead have improved IQ, but that finding was limited to children given placebo (Liu et al. 2002). The analysis that yielded that result was modeled closely on that of a previous report (Ruff et al. 1993) in which declining blood lead concentration was associated with increased IQ independent of chelation treatment, because our primary goal was to attempt to replicate their findings. We now suspect that the difference in the results for placebo and succimer groups was caused by unexpected heterogeneity in the relationship at baseline between blood lead and IQ in the placebo and succimer groups. Removing baseline MDI from the model that was previously used reduced the difference between placebo and succimer groups. In neither analysis did we see evidence in the overall group or in the placebo group that blood lead at 2 years of age determined IQ at 7 years of age. Nevertheless, modeling two change measures (IQ change and blood lead change) in the same model was complicated by intraindividual tracking in blood lead concentration and IQ over time as well as correlation between baseline lead and baseline IQ (Dietrich et al. 1993; Ernhart 1993). Theoretically, putting baseline IQ in the regression model would yield an estimate of the effect of blood lead concentration change on individual IQ change over time; however, the baseline IQ may be on the causal path. Also, IQ at an early age has less stability (Sternberg et al. 2001), and the use of different IQ test measures at 2 years of age and school age (Liu et al. 2002; Ruff et al. 1993; Tong et al. 1998) may actually introduce more variance. Thus, although it is attractive to look at change in IQ by change in blood lead when considering the effect of an intervention, modeling those changes simultaneously is complex and can produce results that are difficult to interpret. A simpler approach is to model IQ at school age by prior and concurrent blood lead concentration: If higher concurrent blood lead concentrations are associated with lower IQ, then it is plausible that we should attempt to keep blood lead concentrations low.

It is prudent to ponder whether greater association between concurrent blood lead and IQ score at school age was due to reverse causality. This could happen if the lower IQ preceded higher lead concentrations. In this data set, baseline MDI was not associated with blood lead concentration at 7 years, with or without adjustment of baseline blood lead. This supports the findings of the longitudinal Port Pirie study (Tong et al. 1996) and supports the notion that lead exposure causes cognitive deficiency in children within approximately normal intelligence range, but not the reverse, although the association between much lower IQ than seen in these study subjects and later high blood lead through pica cannot be tested here. Another way in which the strengthening association over time may be produced is to have a fixed effect of the higher blood lead concentrations at 2 years of age concentrated in a more restricted range of blood lead concentrations at 7 years of age. In the TLC study, however, the range of blood lead concentration at entry was 24 μg/dL, and the range at 7 years of age was 26 μg/dL, with similar standard deviations. The restricted range of blood lead concentrations in TLC at enrollment might produce an imprecise estimate of the slope of the relationship between IQ and blood lead concentration, which would stabilize as the children led different lives and their blood lead concentrations dispersed. The slope of blood lead concentration at 2 years of age versus IQ at 5 years of age is similar to that seen in the meta-analyses, and the cross-sectional associations are similar to those seen in studies previously reviewed (Pocock et al. 1994; Schwartz 1994). It seems unlikely that the slope would be substantially steeper if more children with lower values at 2 years of age were added, because the range of 10–20 μg/dL is the range most stably estimated from the meta-analyses, and the TLC estimate is already similar to it.

This finding implies that cross-sectional associations seen in older children, such as the school-age children in the National Health and Nutrition Examination Survey data, should not be dismissed as representing a residual from early high lead exposure. Breslau et al. (2001) recently showed that urban Detroit, Michigan, children had declines in IQ from 6 to 11 years of age, whereas suburban children showed no change in the inherently age-adjusted scores. The data suggested that growing up in a racially segregated and disadvantaged community might contribute to a decline in IQ scores in the early school years. However, urban dwelling may serve as a surrogate of individual exposure, such as lead exposure. TLC children had similar communities of origin as the urban children in the study by Breslau et al. (2001). It could be that lead exposure is causally related to this phenomenon, or it could be that factors leading to the families’ inability to improve their housing led to both higher lead levels and lower IQ. In either case, the effect of lead exposure during school age cannot be ruled out entirely. We did not have a direct measure of the degree to which the caregiver interacts with and stimulates the child, such as the Home Observation for Measurement of the Environment score (Caldwell and Bradley 1979).

The strengths of this analysis include the relatively large size of the data set and the degree of testing and quality control that went into the measurement of both blood lead and IQ. The longitudinal nature of these data is a requirement for this kind of analysis, and the TLC study enjoyed remarkably high retention rates for a longitudinal study among relatively disadvantaged families.

Children in the TLC study had blood lead concentrations high enough to be eligible for a clinical trial of drug therapy. These blood lead concentrations are considerably higher than those of most children in the United States today, even children in poverty (Pirkle et al. 1998). Although it may be that the relationships among these variables are qualitatively different in most children whose blood lead levels never reach 20 μg/dL, this is unlikely because the estimates from this study are well within the range of reported lead effects. If concurrent blood lead remains important until school age for optimum cognitive development, and if 6- and 7-year-olds are as or more sensitive than 2-year-olds, then the difficulties in preventing lead exposure are magnified, but the potential benefit of prevention is greater.

## Figures and Tables

**Table 1:** Blood lead concentrations and IQ scores of TLC children.

Variable	No.	Mean ± SD	Age at tests [years (mean ± SD)]
Blood lead concentration (μg/dL)			
Baseline (2 years of age)	780	26.2 ± 5.1	2.0 ± 0.5
5 years of age	731	12.0 ± 5.2	5.0 ± 0.5
7 years of age*^a^*	622	8.0 ± 4.0	7.0 ± 0.2
Peak from baseline to 7 years of age	780	30.6 ± 6.6	2.2 ± 0.7
Average from baseline to 5 years of age	731	17.0 ± 5.0	
Average from baseline to 7 years of age	622	14.4 ± 4.6	
IQ score			
MDI at baseline	765	82.2 ± 13.7	2.0 ± 0.5
IQ at 5 years of age	727	80.6 ± 13.3	5.0 ± 0.5
IQ at 7 years of age	644	86.7 ± 13.3	7.1 ± 0.2

a One child with a blood lead concentration of 50.8 μg/dL was excluded from the entire analysis.

**Table 2:** Adjusted estimates (95% confidence intervals) of the effect of a change of 10 μg/dL in five measures of lead exposure on change on IQ test scores measured at 2, 5, and 7 years of age.

	Independent variable: blood lead concentration (per 10-μg/dL increment)*^a^*				
Outcome variable:IQ score (points)	2 years of age	5 years of age	7 years of age	Peak*^b^*	Average*^c^*
MDI at baseline	−2.9 (−4.7 to −1.0)				
IQ at 5 years of age	−2.3 (−4.1 to −0.5)	−3.5 (−5.3 to −1.7)			−2.9 (−4.8 to −1.0)
IQ at 7 years of age	−1.1 (−2.9 to 0.7)	−2.9 (−4.8 to −1.1)	−5.4 (−7.8 to −2.9)	−0.7 (−2.1 to 0.7)	−3.3 (−5.4 to −1.1)

a Each combination of outcome and independent variables was modeled separately; all were adjusted for clinic center, race, sex, language, parent’s education, parent’s employment, single parent, caregiver’s IQ, and exact age at blood lead concentration measurement.

b Peak blood lead concentration between baseline and 7 years of age.

c Average blood lead concentration from baseline to 5 years of age for IQ at 5 years of age, and average blood lead concentration from baseline to 7 years of age for IQ at 7 years of age.

**Table 3:** Adjusted estimates (95% confidence intervals) of the effect of a 10-μg/dL change in prior and concurrent blood lead concentrations on IQ test scores at 5 and 7 years of age.

		Independent variable: blood lead concentrations (per 10-μg/dL increment)*^a^*		
Outcome variable:IQ score	Additional adjustment for baseline MDI or 5-year IQ score	2 years of age	5 years of age	7 years of age
IQ at 5 years of age	None	−1.2 (−3.1 to 0.7)	−2.9 (−4.9 to −0.9)	
	Baseline MDI	−0.1 (−1.8 to 1.5)	−2.4 (−4.1 to −0.7)	
IQ at 7 years of age	None	0.1 (−1.8 to 2.0)		−5.0 (−7.6 to −2.4)
	Baseline MDI	0.4 (−1.4 to 2.1)		−3.8 (−6.2 to −1.4)
	None		−1.2 (−4.1 to 1.7)	−3.9 (−7.4 to 0.0)
	5-year IQ		1.7 (−0.3 to 3.6)	−3.7 (−6.3 to −1.1)

a Each row of results was from separate models; all were adjusted for clinic center, race, sex, language, parent’s education, parent’s employment, single parent, caregiver’s IQ, and exact age at both blood lead measurements.

**Table 4:** Adjusted estimates of regression coefficients (95% confidence intervals) of categorical blood lead concentrations in models for IQ scores.

			Outcome variable			
			IQ at 5 years*^b^*		IQ at 7 years*^b^*	
Category of blood lead concentration (μg/dL)*^a^*		No.	Mean score	Comparison	Mean score	Comparison
2 years	5 years					
< 24.9	< 11.4	227	83.7	Referent		
< 24.9	≥11.4	137	78.8	−2.9 (−5.8 to 0.1)		
≥24.9	< 11.4	138	82.4	0.4 (−2.5 to 3.3)		
≥24.9	≥11.4	228	77.6	−4.0 (−6.6 to −1.5)		
2 years	7 years					
< 24.9	< 7.2	187			89.3	Referent
< 24.9	≥7.2	114			84.6	−3.6 (−6.4 to −0.7)
≥24.9	< 7.2	121			88.9	−0.0 (−2.8 to 2.7)
≥24.9	≥7.2	195			84.0	−3.7 (−6.2 to −1.3)
5 years	7 years					
< 11.4	< 7.2	244			89.4	Referent
< 11.4	≥7.2	52			85.9	−2.3 (−5.9 to 1.3)
≥11.4	< 7.2	62			88.2	0.3 (−3.1 to 3.7)
≥11.4	≥7.2	255			83.9	−3.8 (−6.0 to −1.6)

a Measured at 2 and 5 years of age, 2 and 7 years of age, and 5 and 7 years of age.

b Adjusted for clinic center, race, sex, language, parent’s education, parent’s employment, single parent, caregiver’s IQ, and exact age at both blood lead measurements.

## References

[b1-ehp0113-000597] Bayley N 1993. Bayley Scales of Infant Development: Manual. 2nd ed. San Antonio, TX:Psychological Corporation.

[b2-ehp0113-000597] Bellinger DC, Stiles KM, Needleman HL (1992). Low-level lead exposure, intelligence and academic achievement: a long-term follow-up study. Pediatrics.

[b3-ehp0113-000597] Breslau N, Chilcoat HD, Susser ES, Matte T, Liang KY, Peterson EL (2001). Stability and change in children’s intelligence quotient scores: a comparison of two socioeconomically disparate communities. Am J Epidemiol.

[b4-ehp0113-000597] Caldwell B Bradley R 1979. Home Observation for Measurement of the Environment. Little Rock, AR:University of Arkansas.

[b5-ehp0113-000597] Canfield RL, Henderson CR, Cory-Slechta DA, Cox C, Jusko TA, Lanphear BP (2003). Intellectual impairment in children with blood lead concentrations below 10 microg per deciliter. N Engl J Med.

[b6-ehp0113-000597] Dietrich KN 1995 A higher level of analysis: Bellinger’s, interpreting the literature on lead and child development Neurotoxicol Teratol 17 223 225249–251. 7542730 10.1016/0892-0362(94)00086-s

[b7-ehp0113-000597] Dietrich KN, Berger OG, Succop PA, Hammond PB, Bornschein RL (1993). The developmental consequences of low to moderate prenatal and postnatal lead exposure: intellectual attainment in the Cincinnati Lead Study Cohort following school entry. Neurotoxicol Teratol.

[b8-ehp0113-000597] Dietrich KN, Ware JH, Salganik M, Radcliffe J, Rogan WJ, Rhoads GG (2004). Effect of chelation therapy on the neuropsychological and behavioral development of lead-exposed children after school entry. Pediatrics.

[b9-ehp0113-000597] Ernhart CB (1993). Declining blood lead levels and cognitive change in children. JAMA.

[b10-ehp0113-000597] Lanphear BP, Dietrich K, Auinger P, Cox C (2000). Cognitive deficits associated with blood lead concentrations < 10 microg/dL in US children and adolescents. Public Health Rep.

[b11-ehp0113-000597] Liu X, Dietrich KN, Radcliffe J, Ragan NB, Rhoads GG, Rogan WJ (2002). Do children with falling blood lead levels have improved cognition?. Pediatrics.

[b12-ehp0113-000597] Miller DT, Paschal DC, Gunter EW, Stroud PE, D’Angelo J (1987). Determination of lead in blood using electrothermal atomisation atomic absorption spectrometry with a L’vov platform and matrix modifier. Analyst.

[b13-ehp0113-000597] Pirkle JL, Kaufmann RB, Brody DJ, Hickman T, Gunter EW, Paschal DC (1998). Exposure of the U.S. population to lead, 1991–1994. Environ Health Perspect.

[b14-ehp0113-000597] Pocock SJ, Smith M, Baghurst P (1994). Environmental lead and children’s intelligence: a systematic review of the epidemiological evidence. Br Med J.

[b15-ehp0113-000597] Rogan WJ, Dietrich KN, Ware JH, Dockery DW, Salganik M, Radcliffe J (2001). The effect of chelation therapy with succimer on neuropsychological development in children exposed to lead. N Engl J Med.

[b16-ehp0113-000597] Ruff HA, Bijur PE, Markowitz M, Ma YC, Rosen JF (1993). Declining blood lead levels and cognitive changes in moderately lead-poisoned children. JAMA.

[b17-ehp0113-000597] Schwartz J (1994). Low-level lead exposure and children’s IQ: a meta-analysis and search for a threshold. Environ Res.

[b18-ehp0113-000597] Silverstein AB (1985). Two- and four-subtest short forms of the WAIS-R: a closer look at validity and reliability. J Clin Psychol.

[b19-ehp0113-000597] Sternberg RJ, Grigorenko EL, Bundy DA (2001). The predictive value of IQ. Merrill Palmer Q.

[b20-ehp0113-000597] TLC (1998). The Treatment of Lead-Exposed Children (TLC) trial: design and recruitment for a study of the effect of oral chelation on growth and development in toddlers. Paediatr Perinat Epidemiol.

[b21-ehp0113-000597] TLC (2000). Safety and efficacy of succimer in toddlers with blood lead levels of 20–44 microg/dL. Treatment of Lead-Exposed Children (TLC) trial group. Pediatr Res.

[b22-ehp0113-000597] Tong S, Baghurst P, McMichael A, Sawyer M, Mudge J (1996). Lifetime exposure to environmental lead and children’s intelligence at 11–13 years: the Port Pirie Cohort Study. Br Med J.

[b23-ehp0113-000597] Tong S, Baghurst PA, Sawyer MG, Burns J, McMichael AJ (1998). Declining blood lead levels and changes in cognitive function during childhood: the Port Pirie Cohort Study. JAMA.

[b24-ehp0113-000597] Wasserman GA, Liu X, Lolacono NJ, Factor-Litvak P, Kline JK, Popovac D (1997). Lead exposure and intelligence in 7-year-old children: the Yugoslavia Prospective Study. Environ Health Perspect.

[b25-ehp0113-000597] Wasserman GA, Liu X, Popovac D, Factor-Litvak P, Kline J, Waternaux C (2000). The Yugoslavia prospective lead study: contributions of prenatal and postnatal lead exposure to early intelligence. Neurotoxicol Teratol.

[b26-ehp0113-000597] Wechsler D 1981. Wechsler Adult Intelligence Scale for Adults, Revised. San Antonio, TX:Psychological Corporation.

[b27-ehp0113-000597] Wechsler D 1989. The Wechsler Preschool and Primary Scales of Intelligence-Revised. San Antonio, TX:Psychological Corporation.

[b28-ehp0113-000597] Wechsler D 1991. Wechsler Intelligence Scale for Children-III. San Antonio, TX:Psychological Corporation.

